# Prognostic Value of Lymphocyte–C-Reactive Protein Ratio in Patients Undergoing Radical Cystectomy for Bladder Cancer: A Population-Based Study

**DOI:** 10.3389/fonc.2021.760389

**Published:** 2021-10-28

**Authors:** Houliang Zhang, Yidi Wang, Jinliang Ni, Huajuan Shi, Tao Zhang, Yifan Zhang, Jing Guo, Keyi Wang, Weipu Mao, Bo Peng

**Affiliations:** ^1^ Department of Urology, Shanghai Putuo District People’s Hospital, Tongji University, Shanghai, China; ^2^ Department of Urology, Shanghai Tenth People’s Hospital, School of Medicine, Tongji University, Shanghai, China; ^3^ Department of Obstetrics & Gynecology, Shanghai Tenth People’s Hospital, Tongji University, Shanghai, China; ^4^ Department of Urology, Affiliated Zhongda Hospital of Southeast University, Nanjing, China

**Keywords:** lymphocyte–C-reactive protein ratio, bladder cancer, radical cystectomy, prognosis, nomogram

## Abstract

**Purpose:**

This study aimed to assess the prognostic value of the lymphocyte–C-reactive protein ratio (LCR) in patients with bladder cancer (BCa) who underwent radical cystectomy (RC).

**Materials and Methods:**

BCa patients between 2009 and 2018 were retrieved from our medical center. The predictive value of LCR on survival of BCa patients was evaluated through the Kaplan–Meier survival and receiver operating characteristic (ROC) curves. The multivariate Cox regression results were used for conducting the nomogram, which were further verified by ROC, decision curve analysis (DCA), and calibration curves. Propensity score matching (PSM) was performed to validate our findings.

**Results:**

A total of 201 BCa patients who received RC were included in this study, with 62 (30.8%) patients in the low LCR group and 139 (69.2%) in the high LCR group. Multivariate analysis results revealed that the high LCR group was significantly related to better prognosis and functioned as a prognostic biomarker for overall survival (OS) [hazard ratio (HR) = 0.41, 95% CI, 0.26–0.66; *p* < 0.001] and disease-free survival (DFS) [HR = 0.40, 95% CI, 0.26–0.66; *p* < 0.001]. The nomogram processed better predictive capability and accuracy than TNM stage from ROC results (AUC = 0.754 *vs*. AUC = 0.715), with the confirmation of calibration curves and DCA. The result of PSM confirmed that LCR was significantly correlated with OS and DFS.

**Conclusion:**

Our finding demonstrates that LCR is a novel, convenient, and effective predictor that may provide vital assistance for clinical decision and individualized therapy in BCa patients after RC.

## Introduction

Bladder cancer (BCa) has become a common cancer, ranking ninth in morbidity and 13th in mortality among malignancies worldwide (1). At present, TNM staging is widely used in clinical staging of BCa, in which Tis, Ta, and T1 BCas are collectively considered as non-muscular invasive BCa (NMIBC), and T2 or above BCas are considered as muscular invasive BCa (MIBC) (2). Radical cystectomy (RC) with regional pelvic lymph node dissection (PLND) is the established standard of therapy for MIBC and high-risk NMIBC (3). Despite curative intent, the survival rates are still not satisfactory after RC treatment. The 5-year and 10-year overall survival (OS) rates were 66% and 43%, respectively, for patients who received RC (4). Although TNM staging is one of the most valuable indicators to judge the clinical prognosis, the clinical outcome for patients after RC may vary even with similar stage and grade of BCa. Except for the heterogeneous characteristic of BCa, initial under-staging is also an important risk factor of disease progression and poor outcomes (4, 5). Therefore, it is important and reasonable to seek effective approaches to enhance clinical decision determination and assist clinicians to identify appropriate surgical interventions and treatments for patients during the perioperative course.

To date, a growing body of research has indicated that systemic inflammation *via* host–tumor interactions is closely related to tumor development and metastasis of various malignancies and is considered as the seventh cancer hallmark (6–8). Several studies have found that peripheral blood cells, including lymphocytes, neutrophils, and platelets, may promote the proliferation, migration, and invasion of tumor cells (9); and C-reactive protein (CRP) promotes cancer development with direct correlation to circulating concentrations of vascular endothelial growth factor (VEGF) (10). In view of the above reasons, accumulating studies have combined hematological components of systemic inflammatory responses to develop inflammation-based prognostic scores such as platelet-to-lymphocyte ratio (PLR) (11), neutrophil-to-lymphocyte ratio (NLR) (12), lymphocyte-to-monocyte ratio (LMR) (13), Glasgow prognostic score (GPS) (14), prognostic nutritional index (PNI) (15), and prognostic index (PI) (16) for functioning as prognostic biomarkers in different cancers, including BCa. It was recently reported that the lymphocyte–CRP ratio (LCR), a novel prognostic score based on the preoperative lymphocyte count and CRP, has emerged as an independent indicator of poor prognosis in various cancers, including colorectal cancer (17), gastric cancer (18), and hepatocellular carcinoma (19). However, the potential prognostic value of LCR for patients with BCa undergoing RC is unknown.

Therefore, in this study, we will initially assess the prognostic value of LCR in BCa patients undergoing RC. The relationship between LCR and clinicopathological parameters, OS, and disease-free survival (DFS) was first investigated. Then we constructed a nanogram combining LCR and TNM staging system to improve the prediction of 3- and 5-year survival in BCa patients after RC.

## Patients and Methods

### Patients

A total of 201 BCa patients who underwent RC were reviewed at Shanghai Tenth People’s Hospital between January 2009 and October 2018 in this study. These patients were included according to the following criteria: 1) pathological examination supported the diagnosis of BCa; 2) no other anticancer treatment; and 3) >age 18 years. Patients were excluded if they had any of the following: 1) loss of follow-up; 2) missing data; 3) mental illness; 4) other cancers. All treatments were approved by the Ethics Committee of Shanghai Tenth People’s Hospital, School of Medicine, Tongji University (SHSY-IEC-KY-4.0/18-68/01), and complied with institutional and national guidelines. A total of 153 patients who underwent RC at Zhongda Hospital of Southeast University were included in the external validation.

### Clinical Variables

All the clinical variables were retrieved from the hospital electronic records. **Table 1** describes the clinicopathological features of 201 patients. Clinical variables included demographic data, CRP, comprehensive complication index (CCI), and tumor stage (T stage, N stage, M stage, and tumor grade). The blood sample was obtained from each patient through venipuncture. The calculation of LCR was represented as follows: lymphocyte count (10^9^/L) to serum CRP level (mg/L).

**Table 1:** Clinical characteristics of the patients according to LCR before PSM.

Characteristics	All patientsN = 201	LCR		*p*-Value
		Low LCR	High LCR	
		N = 62	N = 139	
Age, years				0.001
≤65	97 (48.3)	19 (30.6)	78 (56.1)	
>65	104 (51.7)	43 (69.4)	61 (43.9)	
Sex				0.883
Male	174 (86.6)	54 (87.1)	120 (86.3)	
Female	27 (13.4)	8 (12.9)	19 (13.7)	
BMI, kg/m^2^				0.002
≤24	120 (59.7)	47 (75.8)	73 (52.5)	
>24	81 (40.3)	15 (24.2)	66 (47.5)	
CCI				0.222
≤2	126 (62.7)	35 (56.5)	91 (65.5)	
>2	75 (37.3)	27 (43.5)	48 (34.5)	
T stage				0.352
T1	79 (39.3)	20 (32.3)	59 (42.4)	
T2	43 (21.4)	12 (19.4)	31 (22.3)	
T3	41 (20.4)	15 (24.2)	26 (18.7)	
T4	38 (18.9)	15 (24.2)	23 (16.5)	
N stage				0.090
N0	166 (82.6)	47 (75.8)	119 (85.6)	
N+	35 (17.4)	15 (24.2)	20 (14.4)	
M stage				0.366
M0	192 (95.5)	58 (93.5)	134 (96.4)	
M1	9 (4.5)	4 (6.5)	5 (3.6)	
Grade				0.273
Low grade	12 (6.0)	2 (3.2)	10 (7.2)	
High grade	189 (94.0)	60 (96.8)	129 (92.8)	

PSM, propensity score matching; LCR, lymphocyte–C-reactive protein ratio; CCI, comprehensive complication index; BMI, body mass index.

### Patient Follow-Up

After treatments had been completed, all patients who underwent RC were followed up routinely. After discharge, the regularity of follow-up visits was every 3 months for the first 2 years and then every 6 months for the following year. The deadline for follow-up was January 20, 2019, or death. The routine examination of the patient included laboratory test, physical examination, and CT. OS was defined as the date from surgery to death or the last follow-up. DFS was defined as time from surgery to disease recurrence or the last follow-up.

### Statistical Analysis

X-tile was used to determine the optimal cutoff level of LCR based on the receiver operating characteristic (ROC) curve (20). According to the result, OS and DFS were compared by the Kaplan–Meier method, and log-rank tests were used to determine significance. The area under the curve (AUC) of LCR was measured and compared. Patients were allocated to the high LCR group and low LCR group. Multivariate Cox regression models were utilized to identify univariate survival analyses and were performed to calculate the associated hazard ratio (HR) and 95% CI.

Based on the results of multivariate Cox regression models, OS and DFS nomograms for 3- and 5-year survival were generated using R3.2.1 (Institute of Statistics and Mathematics, Vienna, Austria) software. We also used ROC curves and decision curve analysis (DCA) curves to verify the predictive capability and accuracy of the nomogram, respectively. These assessments were validated internally and externally through a bootstrap that contained 1,000 resamples and 10-fold cross-validation. Then the calibration curve was applied to evaluate the accuracy of the nomogram. In calibration curve, if the forecasted values are equivalent to the actual observed values, the curve will land on the ideal 45° line (21). With the use of propensity score matching (PSM) based on eight clinical variables, 57 pairs were ascertained and compared by OS and DFS. All statistical analyses were conducted using IBM SPSS 20.0 software (IBM, USA) and GraphPad Prism8 software (GraphPad Software Inc., La Jolla, CA, USA). *p*-Values less than 0.05 were considered to be statistically significant.

## Results

### Patient Characteristics

As described above, the cutoff level of LCR was 0.0857. The data were shown in **Figure S1**. The clinicopathological features of 201 patients who underwent RC are presented in **Table 1**. The gender ratio was 174 (86.6%) males to 27 (13.4%) females. Sixty-two (31%) patients were stratified into the low LCR group, and 139 (69%) patients were distributed into the high LCR group. After stratification, the low LCR group was older than the high LCR group (age ≥65, 30.6% *vs*. 56.1% *p* = 0.001) and was significantly associated with low body mass index (BMI) (75.8% *vs*. 52.5% *p* = 0.002). Sex, CCI, T stage, M stage, N stage, and grade were similar between the two groups (*p* > 0.05).

### Impact of Lymphocyte–C-Reactive Protein Ratio on Overall Survival and Disease-Free Survival

**Figure 1:**
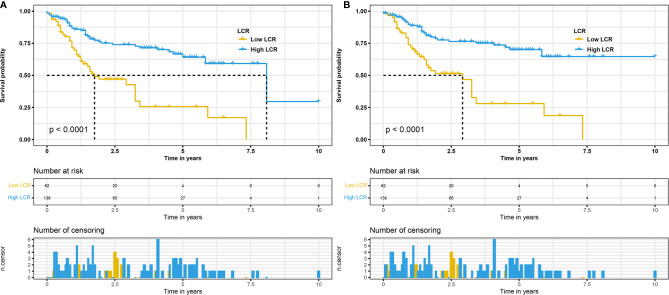
Kaplan–Meier curves for OS in patients with radical cystectomy stratified based on LCR before propensity score matching **(A)**. Kaplan–Meier curves for DFS in patients with radical cystectomy stratified based on LCR before propensity score matching **(B)**. OS, overall survival; LCR, lymphocyte–C-reactive protein ratio; DFS, disease-free survival.

To explore the relationship between LCR and OS with DFS, the Kaplan–Meier curve for patients was used to analyze it, and the data are presented in **Figure 1**. The results revealed that the low LCR group had significantly lower median OS and DFS than the high LCR group. Overall, patients in the low LCR group were significantly correlated with poor OS (*p* < 0.001 **Figure 1A**) and DFS (*p* < 0.001 **Figure 1B**) than the high LCR group.

### Univariate and Multivariate Analyses of Different Clinical Factors

Next, we attempted to elucidate the relationship between different clinical factors and OS with DFS through univariate analysis and multivariate analysis. Univariate analysis revealed that low LCR, M1 stage, higher T stage, and N+ stage were related to poor prognosis (**Tables 2**, **3**). Multivariate analyses indicated that the significant prognostic factors for OS were low LCR status and T stage (**Table 2**). For DFS, the significant prognostic factors for DFS were low LCR status and T stage, too (**Table 3**). As shown in **Table 2**, when T1 and low LCR were used as references, the relative risk for T2 was 2.21 (95% CI, 1.08–4.54; *p* = 0.031), relative risk for T3 was 3.37 (95% CI, 1.73–6.56; *p* < 0.001), relative risk for T4 was 4.71 (95% CI, 2.41–9.21; *p* < 0.001), and relative risk for high LCR was 0.41 (95% CI, 0.26–0.66; *p* < 0.001). For DFS (**Table 3**), the related biomarkers were investigated, and the results indicated that low LCR and T stage were significant prognostic biomarkers. With the use of T1 and low LCR as references again, the relative risk for T2 was 3.99 (95% CI, 1.67–9.54; *p* = 0.002), relative risk for T3 was 5.92 (95% CI, 2.61–13.47; *p* < 0.001), relative risk for T4 was 8.06 (95% CI, 3.51–18.53; *p* < 0.001), and relative risk for high LCR was 0.40 (95% CI, 0.24–0.66; *p* < 0.001).

**Table 2:** Relative risk of overall survival (OS).

Characteristics	Univariate analysis		Multivariate analysis	
	aHR (95% CI)	*p*-Value	aHR (95% CI)	*p*-Value
Age, years				
≤65	Reference		Reference	
>65	1.52 (0.96–2.42)	0.076	–	0.501
Sex				
Male	Reference		Reference	
Female	1.00 (0.51–1.95)	0.995	–	0.867
BMI, kg/m^2^				
≤24	Reference		Reference	
>24	0.78 (0.48–1.26)	0.300	–	0.837
CCI				
≤2	Reference		Reference	
>2	0.92 (0.58–1.47)	0.736	–	0.227
T stage				
T1	Reference		Reference	
T2	2.27 (1.10–4.67)	0.026	2.21 (1.08–4.54)	0.031
T3	3.81 (1.97–7.39)	<0.001	3.37 (1.73–6.56)	<0.001
T4	5.75 (2.96–11.18)	<0.001	4.71 (2.41–9.21)	<0.001
N stage				
N0	Reference		Reference	
N+	3.40 (2.06-5.61)	<0.001	–	0.081
M stage				
M0	Reference		Reference	
M1	2.88 (1.16–7.19)	0.023	–	0.137
Grade				
Low grade	Reference		Reference	
High grade	1.91 (0.69–5.31)	0.217	–	0.846
LCR				
Low	Reference		Reference	
High	0.34 (0.21–0.53)	<0.001	0.41 (0.26–0.66)	<0.001

BMI, body mass index; aHR, adjusted hazard ratio; LCR, lymphocyte–C-reactive protein ratio; CCI, comprehensive complication index.

**Table 3:** Relative risk of disease-free survival (DFS).

Characteristics	Univariate analysis		Multivariate analysis	
	aHR (95% CI)	*p*-Value	aHR (95% CI)	*p*-Value
Age, years				
≤65	Reference		Reference	
>65	1.49 (0.91–2.46)	0.116	–	0.756
Sex				
Male	Reference		Reference	
Female	0.91 (0.43–1.92)	0.807	–	0.998
BMI, kg/m^2^				
≤24	Reference		Reference	
>24	0.65 (0.38–1.10)	0.109	–	0.686
CCI				
≤2	Reference		Reference	
>2	0.82 (0.49–1.37)	0.458	–	0.084
T stage				
T1	Reference		Reference	
T2	4.11 (1.72–9.83)	0.001	3.99 (1.67–9.54)	0.002
T3	6.72 (2.96–15.21)	<0.001	5.92 (2.61–13.47)	<0.001
T4	9.94 (4.35–22.71)	<0.001	8.06 (3.51–18.53)	<0.001
N stage				
N0	Reference		Reference	
N+	3.76 (2.21–6.41)	<0.001	–	0.091
M stage				
M0	Reference		Reference	
M1	3.53 (1.41–8.83)	0.007	–	0.062
Grade				
Low grade	Reference		Reference	
High grade	2.03 (0.64–6.52)	0.232	–	0.878
LCR				
Low	Reference		Reference	0.002
High	0.31 (0.19–0.51)	<0.001	0.40 (0.24–0.66)	<0.001

BMI, body mass index; aHR, adjusted hazard ratio; LCR, lymphocyte–C-reactive protein ratio; CCI, comprehensive complication index.

### Construction of a Nomogram and Validation of Prognostic Efficiency

**Figure 2:**
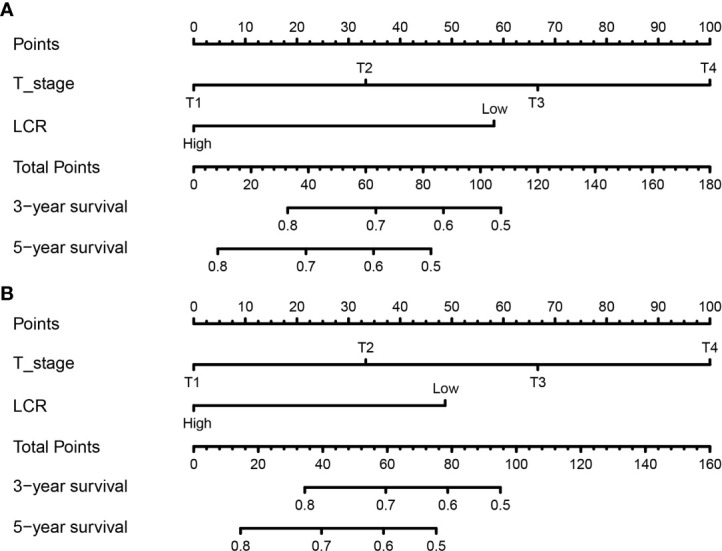
Establishment of nomograms for the prediction of 3- and 5- years OS **(A)** and DFS **(B)** in patients. OS, overall survival; DFS, disease-free survival.

T stage and LCR as the significant prognostic indicators were used to create prognostic nomogram to quantitatively predict OS (**Figure 2A**) and DFS (**Figure 2B**) after RC in BCa patients. The probability of survival for BCa patients who suffered RC within 3 or 5 years can be predicted by the nomogram. Every individual risk factor has a unique score, and a higher total score indicates a worse outcome in the nomogram.

**Figure 3:**
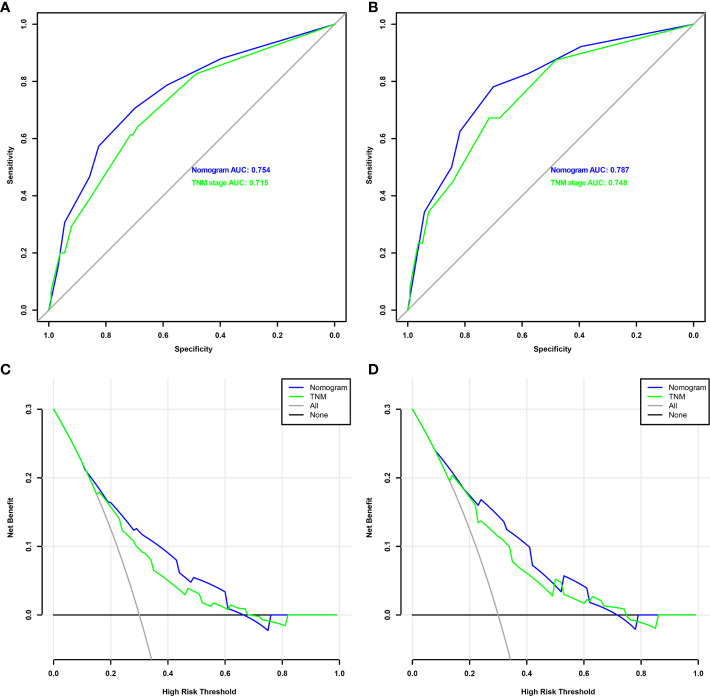
ROC analysis of nomogram and TNM stage to predict the OS **(A)** and DFS **(B)** in patients who underwent radical cystectomy. The decision curve analysis of nomogram and TNM stage for survival benefit in OS **(C)** and DFS **(D)**. ROC, receiver operating characteristic; OS, overall survival; DFS, disease-free survival.

**Figure 4:**
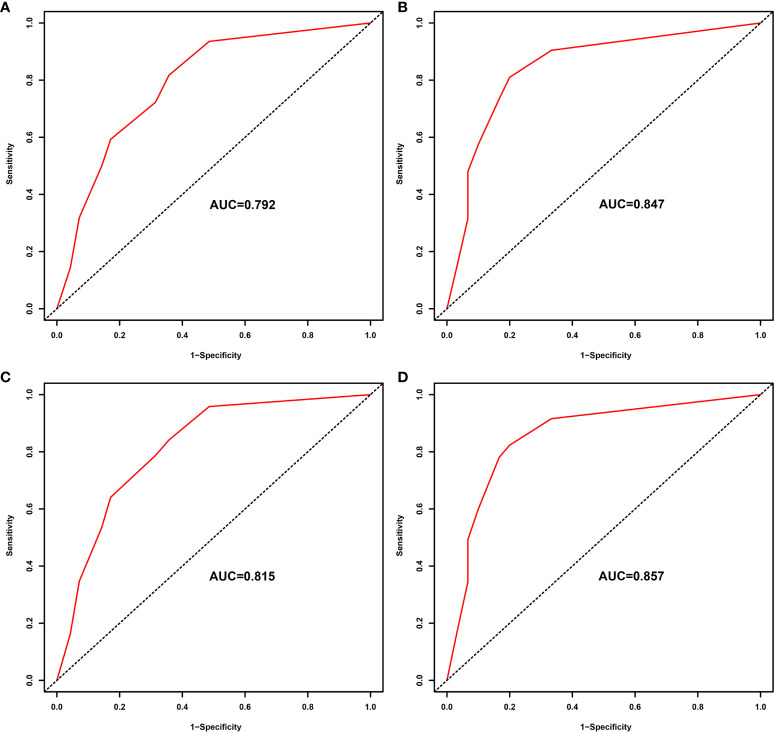
ROC analysis of the prognostic accuracy of nomogram for 3- year overall survival (OS) **(A)**, 5-year OS **(B)**, 3-year DFS **(C)**, and 5-year DFS **(D)**. ROC, receiver operating characteristic; OS, overall survival; DFS, disease-free survival.

**Figure 5:**
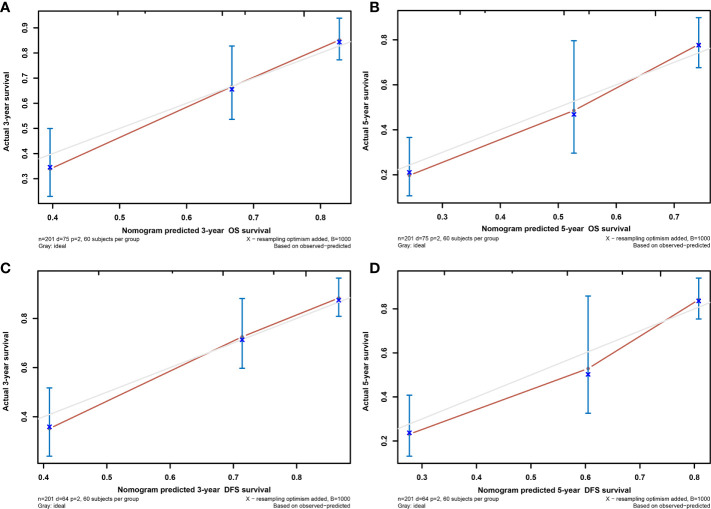
The calibration curves of the nomogram predicting 3-year OS **(A)**, 5-year OS **(C)**, 3-year DFS **(B)**, and 5-year DFS **(D)**. OS, overall survival; DFS, disease-free survival.

We verified predictive capability and accuracy of the nomogram by different methods including ROC curves, DCA curves, and external verification. ROC was used to compare predictive capability of the nomogram with TNM stage. For OS, AUC value of the nomogram (0.754) was larger than that of TNM stage (0.715) (**Figure 3A**). For DFS, AUC value of the nomogram (0.787) was also higher than that of TNM stage (0.748) (**Figure 3B**). DCA as a tool evaluated predictive capability by comparing net benefits (NBs) in different models. The nomogram had more NBs than TNM stage, demonstrating that nomogram had better predictive capability and accuracy (**Figures 3C, D**). We further analyzed 3- and 5-year OS (0.792, 0.847, respectively) (**Figures 4A, B**) and DFS (0.815, 0.857, respectively) (**Figures 4C, D**) of the nomogram, showing that the nomogram was a valid prediction model. The calibration curves suggested excellent consistency between nomogram predictions and actual observations of 3- and 5-year OS and DFS (**Figure 5**). Finally, the calibration curve for the predictive nomogram showed high agreement between the actual probability and predicted probability of BCa in the external validation (**Figure S2**).

**Figure 6:**
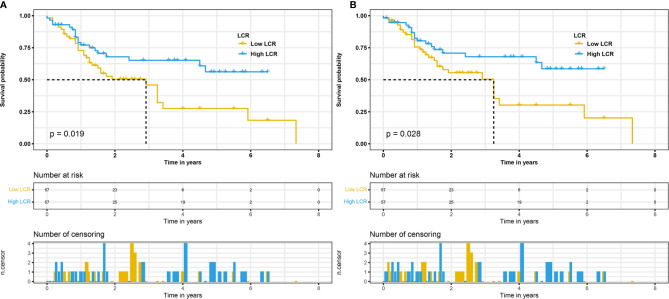
Kaplan–Meier curves for OS in patients with radical cystectomy stratified based on LCR after propensity score matching **(A)**. Kaplan–Meier curves for DFS in patients with radical cystectomy stratified based on LCR after propensity score matching **(B)**. OS, overall survival; LCR, lymphocyte–C-reactive protein ratio; DFS, disease-free survival.

To exclude the interference of other clinical variables, especially TNM stage, we performed PSM on BCa patients who underwent RC. The clinicopathological features of BCa patients after PSM are displayed in **Table 4**. The 114 enrolled patients included 38 (age ≥65, 33.3%) patients and 76 (age <65, 66.7%) patients. The clinical variables including age, BMI, gender, TNM stage, and grade were similar between the high LCR group and low LCR group (all *p* > 0.05). The Kaplan–Meier curves exhibited similar results that patients in the low LCR group were significantly correlated with poor OS (*p* = 0.019, **Figure 6A**) and DFS (*p* = 0.028, **Figure 6B**) than patients in the high LCR group.

**Table 4:** Clinical characteristics of the patients according to LCR after PSM.

Characteristics	All patients	LCR		*p*-Value
		Low LCR	High LCR	
	N = 114	N = 57	N = 57	
Age, years				1.00
≤65	38 (33.3)	19 (33.3)	19 (33.3)	
>65	76 (66.7)	38 (66.7)	38 (66.7)	
Sex				0.568
Male	100 (87.7)	51 (89.5)	49 (86.0)	
Female	14 (12.3)	6 (10.5)	8 (14.0)	
BMI, kg/m^2^				0.677
≤24	82 (71.9)	42 (73.7)	40 (70.2)	
>24	32 (28.1)	15 (26.3)	17 (29.8)	
CCI				1.00
≤2	62 (54.4)	31 (54.4)	31 (54.4)	
>2	52 (45.6)	26 (45.6)	26 (45.6)	
T stage				0.954
T1	42 (36.8)	20 (35.1)	22 (38.6)	
T2	23 (20.2)	12 (21.1)	11 (19.3)	
T3	28 (24.6)	15 (26.3)	13 (22.8)	
T4	21 (18.4)	10 (17.5)	11 (19.3)	
N stage				0.826
N0	87 (76.3)	44 (77.2)	43 (75.4)	
N+	27 (23.7)	13 (22.8)	14 (24.6)	
M stage				0.647
M0	109 (95.6)	54 (94.7)	55 (96.5)	
M1	5 (4.4)	3 (5.3)	2 (3.5)	
Grade				0.154
Low grade	2 (1.8)	2 (3.5)	0 (0.0)	
High grade	112 (98.2)	55 (96.5)	57 (110.0)	

PSM, propensity score matching; LCR, lymphocyte–C-reactive protein ratio; CCI, comprehensive complication index; BMI, body mass index.

## Discussion

In our study, we investigated different clinical variables and prognosis of 201 BCa patients and analyzed the clinical meaning of the novel prognostic biomarker based on lymphocyte to serum CRP (LCR) in our cohort. The results indicated that LCR was significantly related to OS and DFS according to the Kaplan–Meier curves and could be used as a convenient and effective predictive biomarker. Then we built a prognostic nomogram to quantitatively predict 3- and 5-year OS and DFS based on T stage and LCR. The calibration curves were used to verify the predictive capability and accuracy of nomogram. It was supposed that this study was the first to analyze the prognostic value of LCR in BCa patients.

Increasing evidence suggests that systemic inflammation caused by host–tumor interactions is closely associated with the development and metastasis of various malignancies. Thereby, tumor prognosis could be affected by systemic inflammation. CRP as the main inflammatory biomarker was used to assess disease activity in several inflammatory diseases and upregulate the expression of VEGF by activating hypoxia-inducible factor-1α (HIF-1α) (10). Previous literatures have shown that lymphocyte count plays a key role in the host immune response to tumors and is considered to reflect the general state of immune function (22, 23). System inflammation biomarkers, including CRP, NLR, and LMR, have been reported to evaluate the prognosis of human cancer (24–26).

LCR, a combination of lymphocyte count and CRP, was correlated with oncological outcomes including recurrence and survival in patients with colorectal cancer (27), intrahepatic cholangiocarcinoma (28), and gastric cancer (29). The function of lymphocyte and CRP may explain the mechanism underlying the relation between low LCR and poor outcomes. As mentioned above, lymphocyte count was related to the host immune response to malignancies (24–26). The reduction of lymphocytes can lead to immune disorders and tumor escape. Inflammatory cells activated inflammatory cytokines and transcription factors, which leads to tumorigenesis and development (30). These cytokines enhance the synthesis of CRP in the liver (31). On the other hand, high CRP was related to sustained inflammation, which may reflect a pro-angiogenic tumor microenvironment, as high CRP upregulated the expression of VEGF permitting tumor proliferation and metastasis (10). It had reported that low preoperative LCR levels were significantly correlated with prognostic factors including distant metastases and lymph node metastases and was a new biomarker for early complications in patients with gastrointestinal cancer (32). Recently, many scholars questioned the prognostic reliability and availability of TNM stage (33). Thus, additional modifications are needed to help improve the prognostic stratification of BCa patients. Based on univariate analysis and PSM results, LCR and T stage were the independent biomarkers for OS and DFS in BCa patients. Then a nomogram conducted used LCR in conjunction with T stage. In comparison with classical TNM stage, the nomogram contained higher AUC values for OS and DFS based on ROC curve. DCA and calibration curves further proved the accurate predictive performance of the nomogram.

This study still had several limitations. Firstly, our study was single central research, which would be prone to selection bias. Secondly, the number of samples was still insufficient and should be expanded to increase the credibility of results. Thirdly, the present work was a retrospective study, which should be verified by the prospective studies. In conclusion, LCR as the reliable and readily accessible preoperative PI is capable of predicting BCa patient’s prognosis. The nomogram can effectively forecast the survival of BCa patients compared with classical TNM stage and provide vital evidence for clinical decision and individualized therapy.

## Data Availability Statement

The raw data supporting the conclusions of this article will be made available by the authors, without undue reservation.

## Ethics Statement

The studies involving human participants were reviewed and approved by the Ethics Committee of Shanghai Tenth People’s Hospital, School of Medicine, Tongji University (SHSY-IEC-KY-4.0/18-68/01). The patients/participants provided their written informed consent to participate in this study.

## Author Contributions

Conception and design: TZ, HZ, and JG. Administrative support: JN, KW, and BP. Provision of study materials or patients: YW, YZ, and HS. Collection and assembly of data: WM, JG, and HZ. Data analysis and interpretation: WM, KW, and HZ. All authors contributed to the article and approved the submitted version.

## Funding

The Shanghai Association for Science and Technology Commission (Grant No. 19140905700) supported this study. This work was supported by the National Natural Science Foundation of China (Grant Nos. 81870517; 32070646) and Science and Technology Innovation Project of Putuo District Health Commission (Grant No. ptkwws201916).

## Conflict of Interest

The authors declare that the research was conducted in the absence of any commercial or financial relationships that could be construed as a potential conflict of interest.

## Publisher’s Note

All claims expressed in this article are solely those of the authors and do not necessarily represent those of their affiliated organizations, or those of the publisher, the editors and the reviewers. Any product that may be evaluated in this article, or claim that may be made by its manufacturer, is not guaranteed or endorsed by the publisher.

## Supplementary Material

Supplementary Figure 1 Determination of the optimal cut-off value for LCR based on the ROC analysis.

Supplementary Figure 2 Calibration curve had good agreement between the actual and predicted probability of Bca.
