# Supplementary figures and images for: Prognostic Value of Lymphocyte–C-Reactive Protein Ratio in Patients Undergoing Radical Cystectomy for Bladder Cancer: A Population-Based Study
Source: Front Oncol. 2021 Oct 28;11:760389. doi: 10.3389/fonc.2021.760389 (PMC8581644; doi:10.3389/fonc.2021.760389)

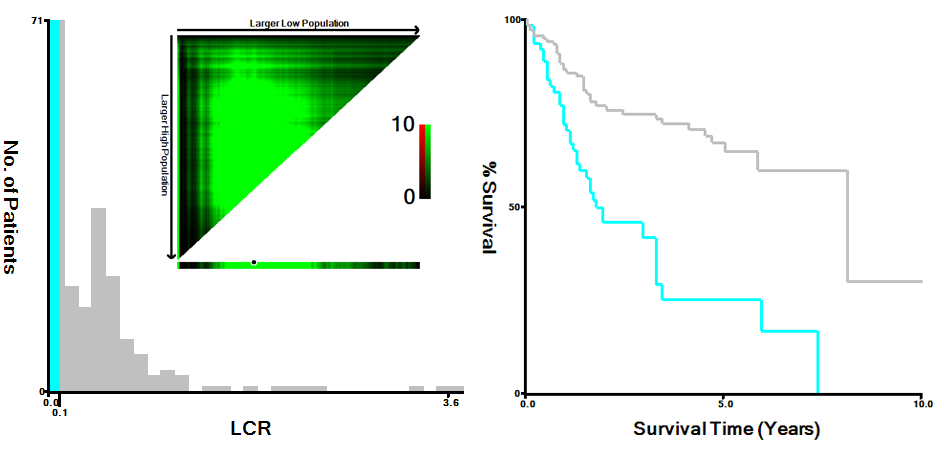

Supplement: Supplementary Figure 1 — Determination of the optimal cut-off value for LCR based on the ROC analysis. [file Image_1.tif]

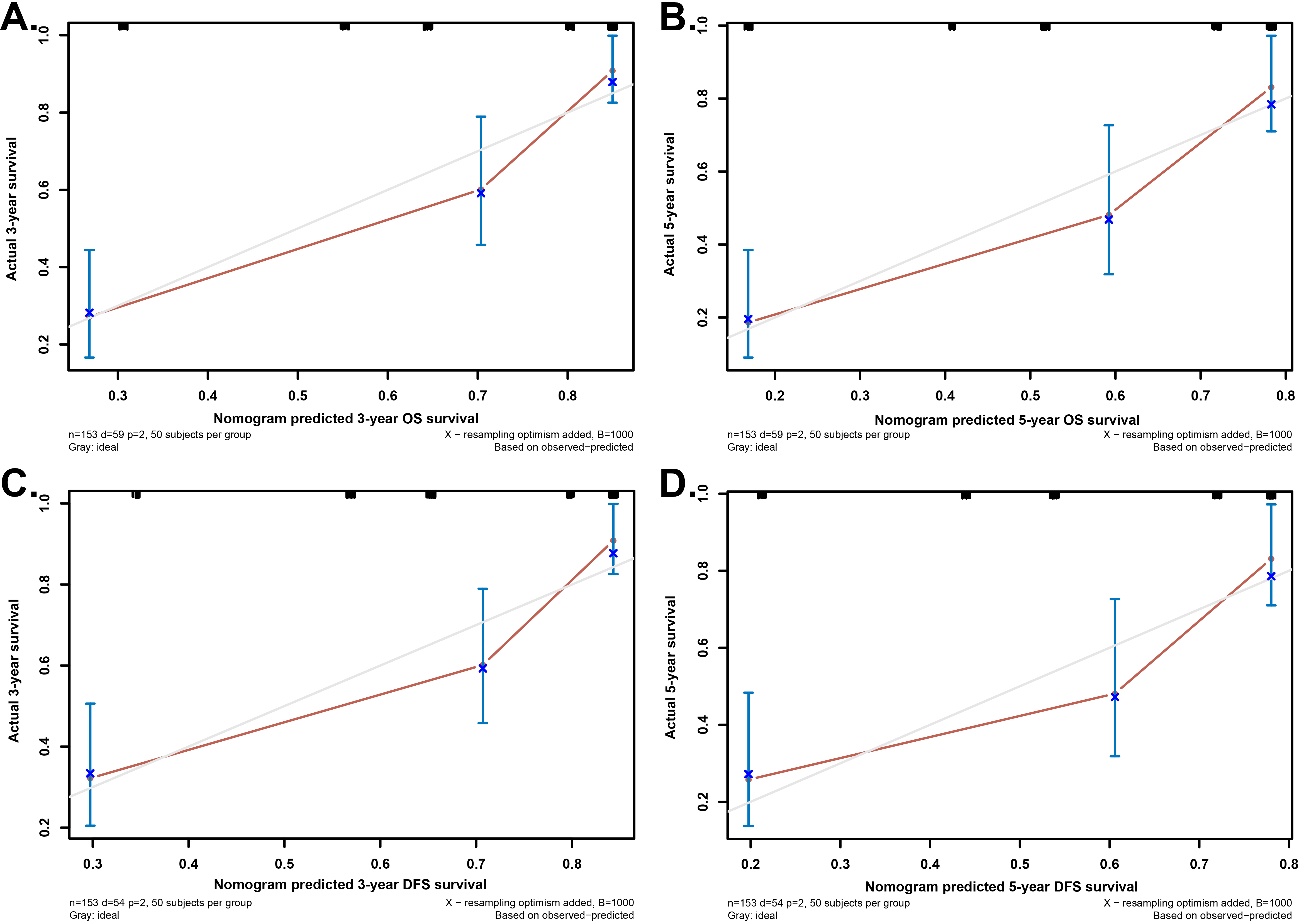

Supplement: Supplementary Figure 2 — Calibration curve had good agreement between the actual and predicted probability of Bca. [file Image_2.tif]
